# Photocatalytic Degradation of Sulfamethoxazole, Nitenpyram and Tetracycline by Composites of Core Shell g-C_3_N_4_@ZnO, and ZnO Defects in Aqueous Phase

**DOI:** 10.3390/nano11102609

**Published:** 2021-10-04

**Authors:** Godfred Kwesi Teye, Jingyu Huang, Yi Li, Ke Li, Lei Chen, Williams Kweku Darkwah

**Affiliations:** 1 Key Laboratory of Integrated Regulation and Resource Development on Shallow Lakes of Ministry of Education, College of Environment, Hohai University, No. 1, Xikang Road, Nanjing 210098, China; teyegodfred@hhu.edu.cn (G.K.T.); w.darkwah@unsw.edu.au (W.K.D.); 2 Department of Civil Engineering, Jilin Jianzhu University, Changchun 130118, China; like_student@163.com (K.L.); cheniei.jiae@hotmail.com (L.C.); 3 School of Chemical Engineering, UNSW Sydney, Sydney, NSW 2052, Australia

**Keywords:** core shell g-C_3_N_4_@ZnO, ZnO defects photocatalysts, nitenpyram, tetracycline, sulfamethoxazole

## Abstract

The synthesis of photocatalysts with high charge separation and transfer efficiency are of immense significance in the process of using photocatalysis technology for wastewater treatment. In this study core shell g-C_3_N_4_@ZnO, and ZnO defects photocatalysts presented an improved morphology in its characterization using techniques such as SEM, DRS, PL, MS, EIS, and XRD, and enhanced photodegradation of sulfamethoxazole, Nitenpyram and Tetracycline. Different composites were obtained as confirmed by the various characterization techniques studied, including core shell g-C_3_N_4_@ZnO, and ZnO defects photocatalyst. The synthesized photocatalysts showed high visible light absorption efficiency within a range of ~655 to 420 nm. Core shell g-C_3_N_4_@ZnO, and ZnO defects photocatalysts demonstrated high photocatalytic activity ascribed to high load separation and transition as shown in PL, Photocurrent reaction and EIS. It is understandable that core shell g-C_3_N_4_@ZnO, and ZnO defects photocatalysts have been confirmed to be one of the ultimate promising entrants for photocatalyst scheming.

## 1. Introduction 

As the population grows, the demand and usage of basic social amenities such as water becomes very high. However, water as an essential basic amenity is often contaminated with pollutants in recent years mostly from the origin of Pharmaceutical and Personal Care Products (PPCPs) [1]. Water is an important resource, required for the survival on earth of aquatic life, animals and humans; however, the discharge of wastewater from treatment plants and other non-point sources tend to pollute water bodies and the environment. This makes wastewater a major source of pollution and a burden to society. Precisely, wastewater discharge from chemical industrialized plants, animal production, other forms of agriculture and improper sewerage treatment plants. This wastewater contains distinct concentrations of large organic fragments and pollutants with PPCPs, the origins of which are extremely poisonous and in some cases carcinogenic in nature [1,2,3,4].

The pollutants enter the aquatic environment in their novel or metabolized form through the disposal of expired pharmaceuticals, as well as household wastewater [5,6]. Ref. [7] affirmed that these compounds sometimes enter water resources through wastewaters generated from houses and medical facilities, which contain parent molecules and metabolites of excrements of humans and animals, as well as effluents from pharmaceutical industries [7]. The pharmaceutical industries are progressively producing stable molecules to achieve the desired pharmacological effects. This results in the persistence of pharmaceuticals in the environment, where they are detected in part due to the limited efficiency of Wastewater Treatment Plants (WWTP) processes [8]. The conventional WWTP is considered inefficient in the removal of these pollutants. This is also due to the fact that the traditional municipal WWTPs have not been precisely designed to degrade excreted pharmaceutical compounds and this has been documented as one of the pathways in which these substances get into the aquatic ecology [9]. The traditional treatment methods such as coagulation, sedimentation and biological removal of contaminants of PPCPs origins in wastewater are mostly considered inefficient [8], and therefore, advanced oxidation processes for instant photocatalysis is considered efficient, effective, and a promising green chemistry and sustainable technology [10,11]. Zhang and Lu and other studies postulate that photocatalysis shows its obvious oxidation process in such a way that the semiconductor photocatalyst could degrade pharmaceutical pollutants into inorganic matter without solids and other secondary and atmospheric pollution just under low-cost solar energy [12,13,14].

Several g-C_3_N_4_ and ZnO-loaded composites have been used to enhance the photocatalytic performance to decompose various organic compounds for the purification of water, considered as promising photocatalysts for the degradation of refractory organic pollutants in wastewater [15]. The g-C_3_N_4_ have been chosen for coupling with ZnO due to the polymeric non-toxic and metal free semiconductor with a photosensitive band gap of 2.7 eV, low cost, environmentally friendly and easy to obtain by exfoliation in the form of ultra-thin nanosheets [16]. Graphite carbon nitride (g-C_3_N_4_) is considered a typical layered structure and is also recognized as a non-toxic metal-free semiconductor [17]. g-C_3_N_4_ -based nanostructures are emerging as an epitome for a variety of photocatalytic applications, such as photocatalytic water reduction, oxidation, and degradation of pollutants [18]. The use of photocatalytic degradation techniques for wastewater treatment ensures the decomposition of organic fragments and pharmaceuticals present because of oxygenation using hydroxyl radicals (HO^*^) generated in the reaction environment [19,20].

Although g-C_3_N_4_@ZnO core-shell heterostructures have been reported for photocatalytic applications, to the best of our knowledge, not much attention has been paid to the detailed relationship between photocatalytic degradation of sulfonamides and tetracycline by the composites of core shell g-C_3_N_4_@ZnO. It is against this background that we investigated the core shell g-C_3_N_4_@ZnO, and the ZnO defects photocatalysts and presented an improved morphology in its characterization using techniques such as SEM, DRS, PL, MS, EIS, and XRD, and enhanced photodegradation of sulfamethoxazole, Nitenpyram and Tetracycline, which then contribute to scientific knowledge along with the existing ones on advanced photocatalytic mechanisms and technology used in wastewater treatment. The design of this nanostructured photocatalysts have been considered to be an effective approach in extending the light-responsive range and to enhance the photo-generated carriers’ separation rate [12]. This study hopes to investigate the effectiveness of composites of core shell g-C_3_N_4_@ZnO for photocatalytic degradation of pharmaceutical residues in wastewater.

## 2. Experimental 

### 2.1. Materials 

Zn (CH_3_COO)_2_·2H_2_O, Citric acid, Methanol, Graphitic Carbon Nitride(g-C_3_N_4_). The materials and chemicals used for this experiment were purchased from Tianjin Xintong Fine Chemicals Company Limited, Tianjin, Hebei Province, P. R. China. All the chemicals used were of pure analytical grade and were used without further treatment in an as-received condition. The whole experiment used de-ionized water.

#### 2.1.1. Preparation of g-C_3_N_4_

Synthesis of g-C_3_N_4_:

To prepare g-C_3_N_4_ nanosheets, 6 g of melamine powder and 15 g ammonium chloride were fully mixed. NH_4_Cl acted as supplementary material, providing a dynamic gas template and facilitating the formation of ultrathin graphene-like g-C_3_N_4_. The mixture was calcined at 550 °C for 4 h in a muffle furnace at a heating rate of 2 °C min^−1^. The prepared g-C_3_N_4_ was further ultrasonically exfoliated for 1 h. The suspension was finally centrifuged and washed with deionized water and ethyl alcohol to remove impurities. The resulting yellow products were collected for further use [16].

#### 2.1.2. Preparation of ZnO

ZnO-stearic acid Nano composite was synthesized as described by [14,21]. In a typical procedure, ZnSO_4_ (1 mol L^−1^ (18.317 g in 100 mL of distilled water)) was mixed with 8.23 g of Na_2_CO_3_ (1 mol L^−1^)/NaOH (1 mol L^−1^) (1:1) to allow a ZnO hydrogel; and stearic acid (SA) 4.0 × 10^−1^ mol L^−1^ was added to the ZnO hydrogel solution. The solution would be placed under stirring at room temperature for 2 h. The suspension was stirred at 60 °C followed by an aging period of 24 h at room temperature. The sample would then be washed and dried at 80 °C for 72 h. 

Analysis in percentage (%) terms that is found would be calculated for ZnO as follows: (C_18_H_36_O_2_)0.38·0.5H_2_O, C: 41.94(41.65), H: 7.20(7.22). 

#### 2.1.3. ZnO Defects

A typical procedure requires that a pure ZnO nanoparticle was prepared by adding an appropriate amount of Zn (CH_3_COO)_2_∙2H_2_O (0.1 mol/L (8 g)) to 50 mL of H_2_O and stirred continuously for 1 h. Citric acid (0.1 mol/L) was then added to the prepared solution. This was immediately followed by the addition of 2.3 mL ethylene glycol (0.1 mol/L) under continuous stirring at a temperature of 80 °C for 2 h. After that, the temperature was increased to 150 °C to obtain a dry gel. The obtained gel was heated at the high temperature of 250 °C to form xerogel. The obtained precursor powder would be conveyed to a preferred sample by calcination at 550 °C for about 3 h in air. 

#### 2.1.4. Preparation of Core Shell g-C_3_N_4_@ZnO Photocatalyst

The beakers and other measuring apparatus were cleansed as part of the lab preparation for the experiment. The substrates were initially cleansed with acetone and cotton wool to remove air dust and related visible contaminants. The substrates were later immersed in K_2_Cr O_7_ solution for 3 h. The mixture was then removed and washed with distilled water to remove precipitation and surface oxides. The substrates were then kept in an oven to air dry for a period of 24 h. An appropriate amount of the as-prepared g-C_3_N_4_ (0.00 g, 0.1 g, 0.2 g, 0.3 g, 0.4 g) was dispersed in 50 mL methanol, and ultrasonicated for 1 h to totally disperse the nanosheets. Then, 0.804 g of Zn (CH_3_COO)_2_·2H_2_O was added to the prepared solution under continuous magnetic stirring at 80 °C for 2 h. 20 mL of H_2_O was gently added to the mixture, and then 0.7 g of citric acid was also added to the solution. The temperature of the mixture was adjusted to 90 °C to obtain the precursor powder samples. The obtained powder was calcinated at 550 °C for 3 h in air using a muffle furnace according to [21]. The product was collected and ground into powder in an agate mortar for further use. 

### 2.2. Characterization

X-ray diffractograms (XRD, Rigaku, SmartLab) were used within the range 10–80° 2θ to analyze the phase purity and crystallite size of the as-synthesized photocatalysts. The morphologies and microstructures were realized using electron transmission microscopy and high-resolution electron transmission microscopy (SEM). A spectrophotometer (Thermo fisher Evolution 220) recorded the UV-Vis diffuse reflectance spectra (DRS).

Transient photocurrent response measurements were performed on an electrochemical system CHI-660E (Chenhua, Shanghai, China) with a standard three -Na_2_SO_4_ solution electrode system (0.2 mol/L) to evaluate the electrical properties of the sample. The platinum electrode and the Ag/AgCl saturated electrode were respectively used as a counter electrode and a reference electrode. 0.020 g of photocatalyst was spread in 3 mL of ethanol with ultrasonic treatment to prepare the working electrode, and the mixture solution was then dip-coated onto an ITO glass working electrode.

To prepare the working electrode, 0.020 g of the photocatalyst was distributed with ultrasonic treatment in 3 mL of ethanol, and the mixture solution was then dip-coated onto an electrode working in ITO glass. The catalyst’s photoluminescence spectroscopy (PL) was measured using the FLS1000 transient fluorescence spectrometer from Edinburgh-state. A 450 W ozone-free xenon lamp with an excitation wavelength of 425 nm was the source of the excitement.

#### Photocatalytic Activity Evaluation

In short, 0.025 g of photocatalysts were suspended in aqueous solutions of Tetracycline (TC), Rhodamine B(RhB) and Nitenpyram (NTP) (50 mL, 10 mg L^−1^) for the degradation of oxcarbazepine (OXC). The suspension was stirred in the dark for 30 min before light irradiation to complete the photocatalyst’s surface adsorption—the organic contaminant desorption equilibrium and dissolved oxygen. A 300 W Xe lamp with a 420 nm filter under magnetic stirring was then irradiated to the suspension. The current for work had been set at 15 A. The suspension was removed from the reaction vessel at a given time interval, and the photocatalyst was removed by centrifugation. Finally, a UV vis-spectrophotometer was used to screen the concentrations of pollutants in a solution. 

The measured absorbance intensities were converted into the reduction ratio calculated using the following expression at different illumination times:

(1) $$\mathrm{Reduction}\;\mathrm{ratio}=\frac{C}{C_{0}}=\left(\frac{\;A_{0}-A_{t}}{A_{0}}\right)\times 100\;\;\;$$

where *A*_0_ and *A*_t_ are the absorbance intensities when illuminated and *t* min, respectively.

Recycling experiments were conducted under the same scenario. 1 mM of ammonium oxalate as the scavenger holes, 1 mM of tert-butanol (TBA) was used in the trapping experiments as the radical hydroxyl scavenger, and 1 mM of p-benzoquinone (BQ) as the radical superoxide scavenger [22,23,24,25]. 

## 3. Results and Discussions 

**Figure 1:**
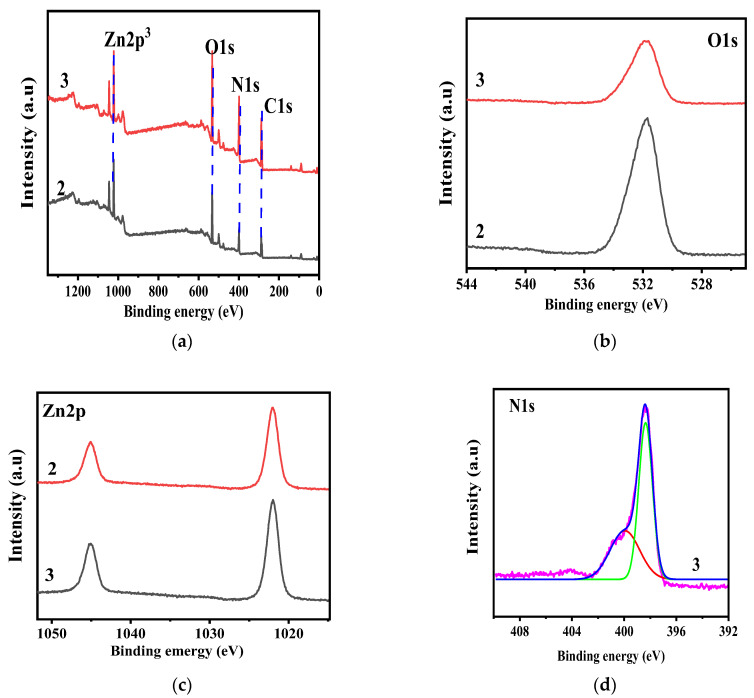
Survey XPS spectra of C_3_N_4_/ZnO, (**a**) and high resolution XPS spectra of (**b**) O 1s (**c**) Zn 2p (**d**) N 1s.

XPS was performed to investigate the chemical composition, chemical state, and electronic structure of the prepared composite nanorods. Figure 1 reveals the XPS spectra of C_3_N_4_/ZnO, which confirms the presence of Zn, N, O, and C and the successful phase transformation of Zn(CH_3_COO)_2_·2H_2_O to C_3_N_4_/ZnO up to ZnO as the calcination temperature and concentration of the nanoparticle increases. The property of the material revealed by the XPS is mainly in line with the literature [26]. 

**Figure 2:**
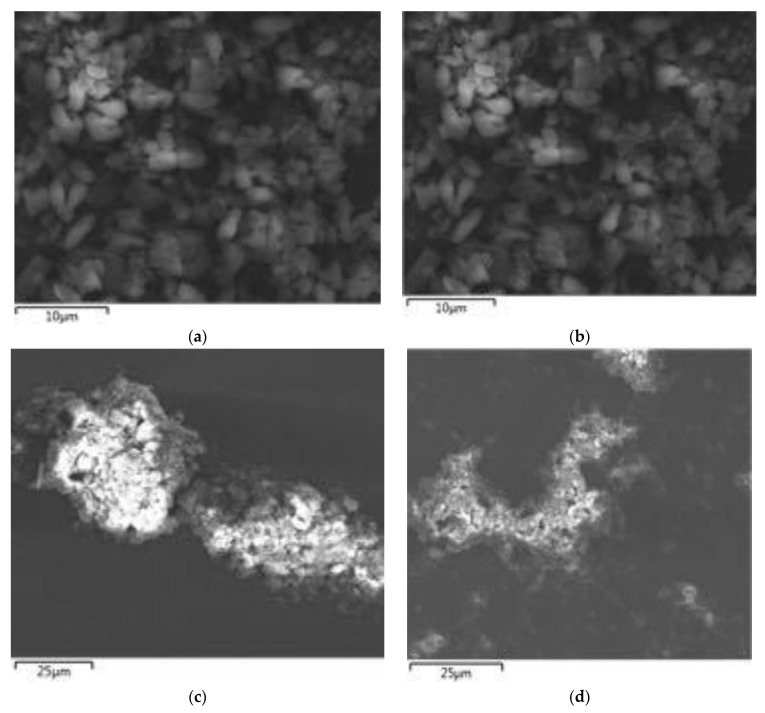
EDX images of (**a**) C_3_N_4_/ZnO—0.1 g (**b**) C_3_N_4_/ZnO—0.2 g (**c**) C_3_N_4_ZnO—0.3 g (**d**) C_3_N_4_/ZnO—0.4 g.

**Figure 3:**
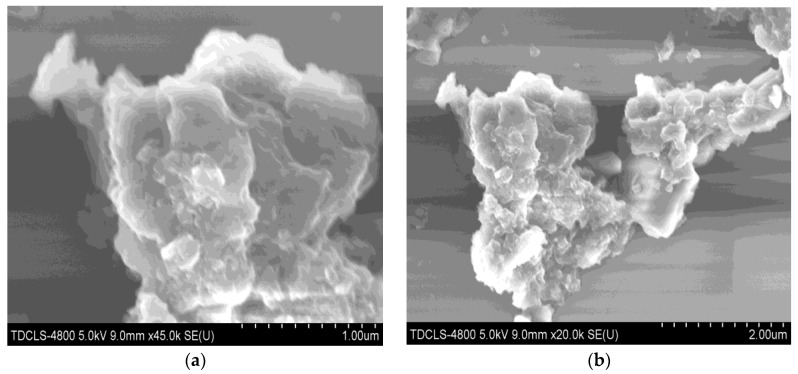

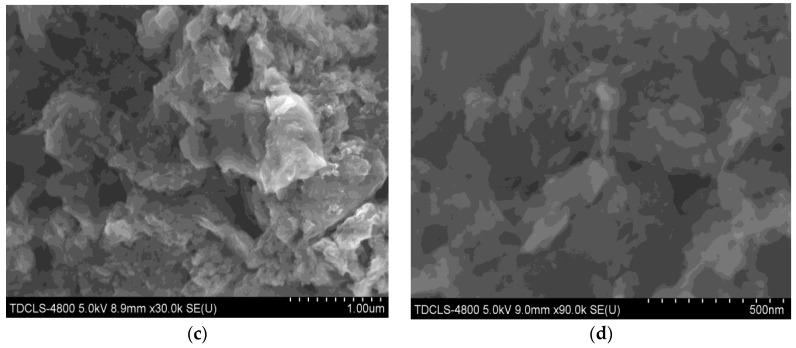
The low magnified SEM and high magnified SEM images of (**a**) C_3_N_4_/ZnO—0.1 g (**b**) C_3_N_4_/ZnO—0.2 g (**c**) C_3_N_4_/ZnO—0.3 g (**d**) C_3_N_4_/ZnO—0.4 g.

The chemical analysis of the prepared sample based on the EDX analysis showed that the Zn: O: C: N, k -ratio is 0.44979: 0.06337: 0.01732: 0.00820, which is close to the theoretical relation. The picture of the as prepared C_3_N_4_/ZnO is shown in Figure 2, the C_3_N_4_/ZnO granules are cylindrical, with a diameter of approximately 2.0 μm and a length of 1.0–2.5 μm (SEM and EDX observed C_3_N_4_/ZnO surface morphology). As shown in Figure 2 and Figure 3, C_3_N_4_/ZnO granules are made up of small particles that form a highly porous composition.

The dispersion of nanorods from C_3_N_4_/ZnO can be seen clearly in Figure 3; thus, from a C_3_N_4_/ZnO composite SEM image (Figure 3). The electron diffraction patterns of selected areas are shown in Figure 3 inset, which also coincides with XRD.

**Figure 4:**
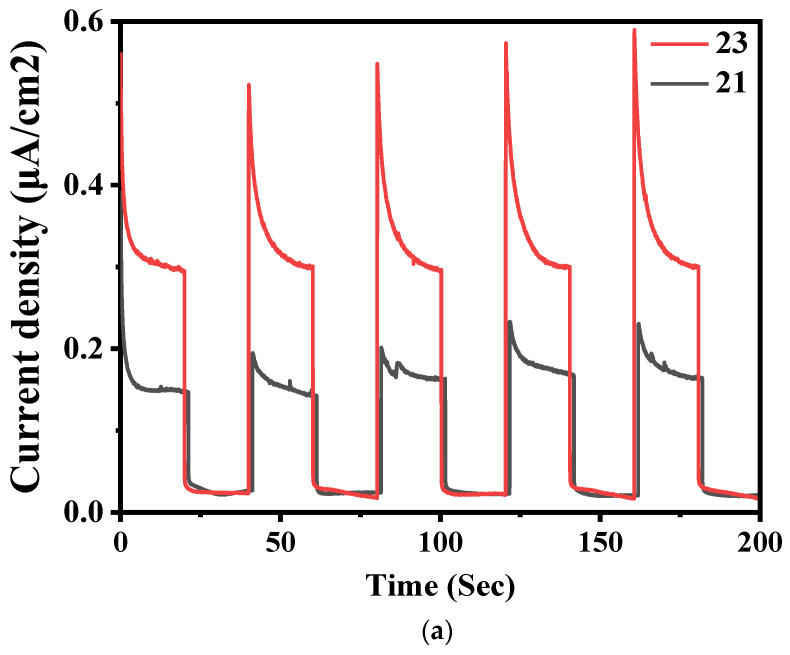

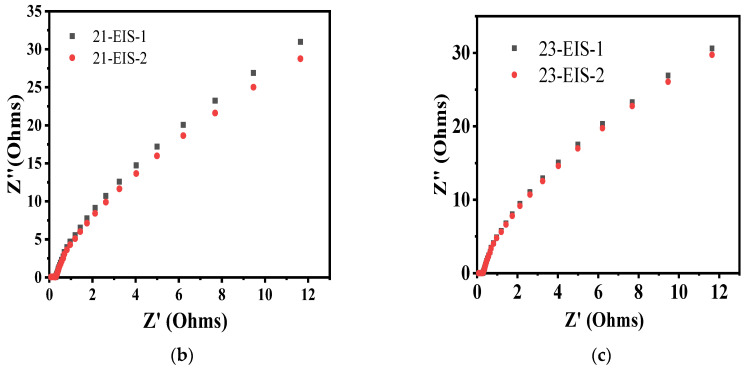
Transient photocurrent response of (**a**) C_3_N_4_/ZnO where 23 is for 0.4 g C_3_N_4_/ZnO; 21 is for 0.3 g C_3_N_4_/ZnO); (**b**) and EIS Nynquist plots of C_3_N_4_/ZnO, 21-EIS-1 stands for 0.4 g C_3_N_4_/ZnO; 21-EIS-2 stands for 0.3 g C_3_N_4_/ZnO; and (**c**) EIS Nynquist plots of ZnO and defect ZnO. 23-EIS-1 belongs to ZnO; 23-EIS-2 belongs to Defect ZnO.

The photocurrent response was used to study the charge carrier transfer of the synthesized samples. As seen in Figure 4a, all the electrodes responded positively to a photocurrent response test with light on and light off. The 0.4 g C_3_N_4_/ZnO (photocatalyst calcined at 550 °C) exhibited higher current response followed by 0.3 g C_3_N_4_/ZnO (photocatalyst calcined at 550 °C) and hence, 0.3 g C_3_N_4_/ZnO (photocatalyst calcined at 550 °C) had a low photocurrent response. The higher photocurrent response peak shows the high charge carrier (electrons and holes) transfer efficiency of the C_3_N_4_/ZnO photocatalyst has an effect in the photocatalytic activity.

Figure 4b,c has a typical electrochemical plot for bare catalyst and its composites. The transfer resistance of charges is directly revealed by arc radius in the Nyquist plot. 0.3 g C_3_N_4_/ZnO and defect ZnO impedance plot has a lower arc radius than pure 0.4 g C_3_N_4_/ZnO and pure ZnO composite catalysts, indicating low load resistance of 0.3 g C_3_N_4_/ZnO and defect ZnO. The 0.4 g C_3_N_4_/ZnO composite acts as a good electron carrier and improves the performance of the photocatalyst. Photogenerated electrons of 0.4 g C_3_N_4_/ZnO could swiftly transfer from the conductive band to ZnO due to low load-transfer resistance (as shown in Figure 4a). A low-frequency linear part indicates ion diffusion/catalyst surface transfer [22].

**Figure 5:**
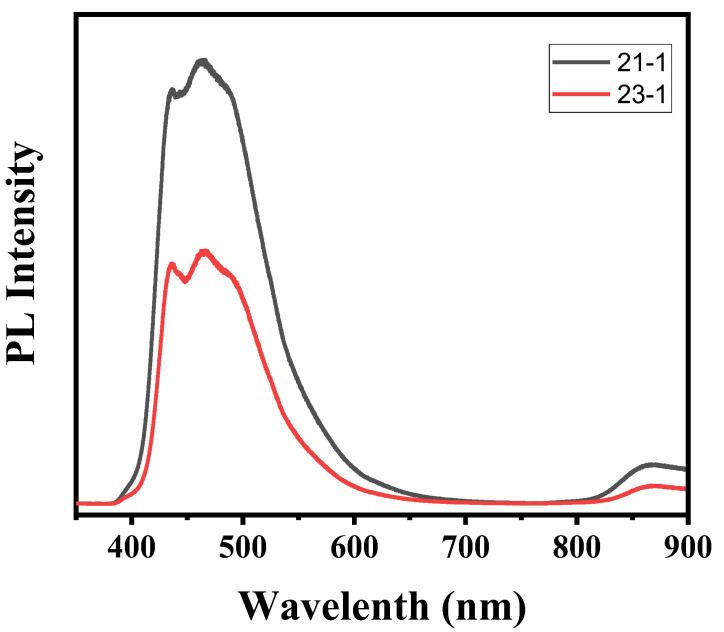
Photoluminescence (PL) spectra of 23 belongs to 0.4 g C_3_N_4_/ZnO; and spectra 21 belongs to 0.3 g C_3_N_4_/ZnO at λ = 510 nm excitation wavelength.

The composites C_3_N_4_/ZnO with various mass fractions are shown in Figure 5. They all turn out to have a related trend narrow band profile when excited at a wavelength of 475 nm. Emission peaks for both compounds were found at around 860 nm. However, when ZnO compounds occur on the outer surface of C_3_N_4_, the PL peak intensity is primarily reduced and then increased. The PL intensity of 0.3 g C_3_N_4_/ZnO is much weaker than 0.4 g C_3_N_4_/ZnO and other composites amongst all as-prepared samples. The reduced emission intensity suggests that recombining charging carriers are effectively self-possessed, which could benefit the ability to photocatalyze. Electrochemical impedance spectroscopy measurements were performed to further study the efficiency of interfacial charge separation.

**Figure 6:**
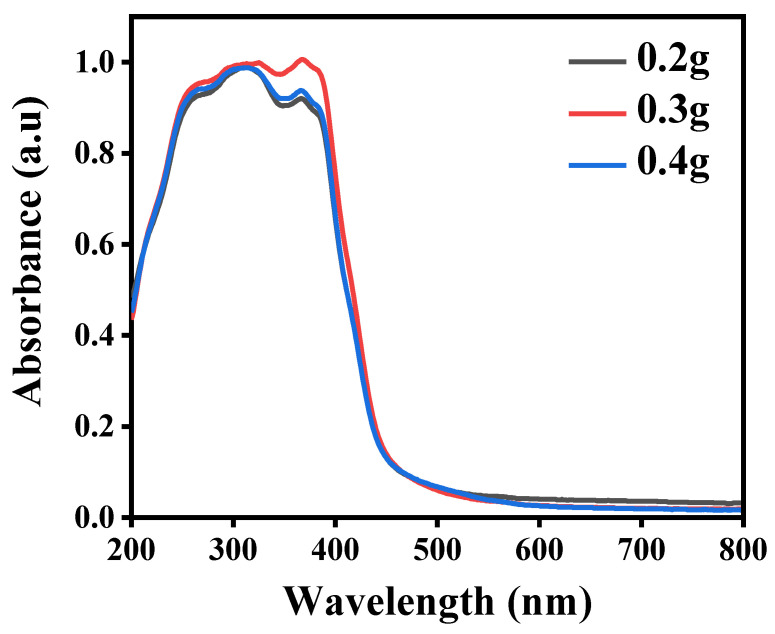
Ultraviolet–visible diffuse reflectance spectroscopy of composites of x g—C_3_N_4_/ZnO.

Figure 6 shows the results of the UV-Vis DRS. The basic edge of the C_3_N_4_/ZnO band is under 338 nm. In contrast, for the visible-light field (>400 nm), both 0.4 g and 0.3 g composites of C_3_N_4_/ZnO has an improved absorption. Both 0.4 g and 0.3 g composites of C_3_N_4_/ZnO have a high 397 nm edge band absorption. The composites of C_3_N_4_/ZnO exhibit strong visibility–light absorption and somehow extend to a near-infrared region. As shown at Figure 6, C_3_N_4_/ZnO and composites of C_3_N_4_/ZnO direct the optical band gap energy (Eg) both 0.4 g, and 0.3 g composites C_3_N_4_/ZnO are measured at 2.70 and 2.75 eV, respectively.

**Figure 7:**
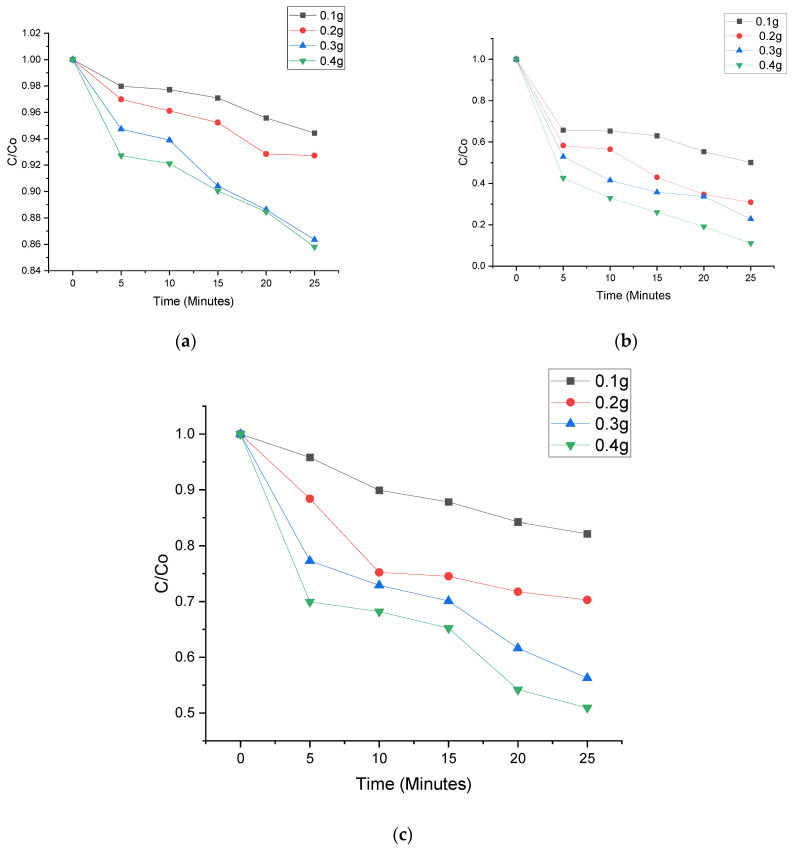
Photo degradation of (**a**) nitenpyram; (**b**) Tetracycline; and (**c**) sulfamethoxazole by Core shell @C_3_N_4_/ZnO.

**Figure 8:**
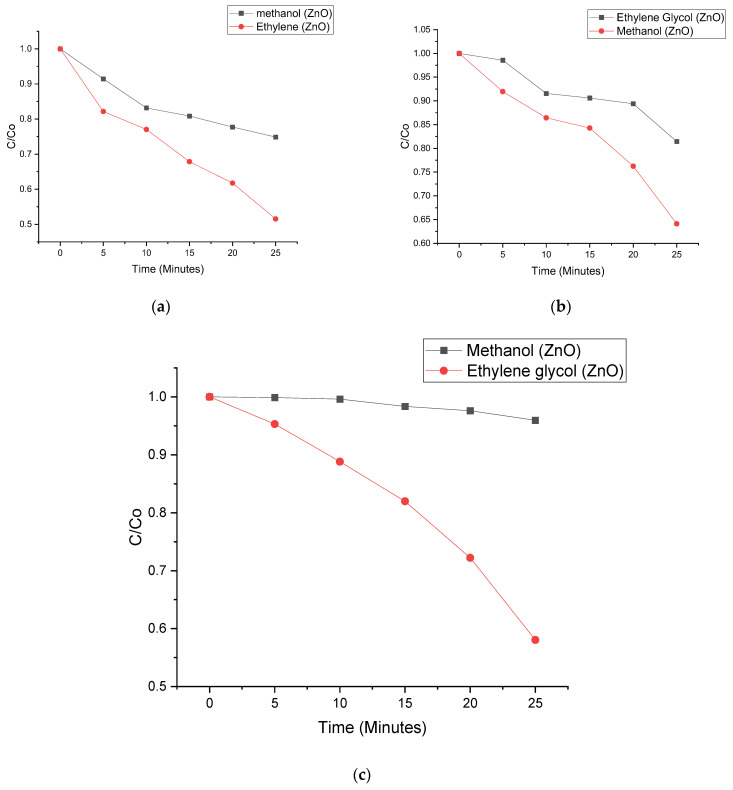
Photo degradation of (**a**) nitenpyram; (**b**) Tetracycline; and (**c**) sulfamethoxazole by methanol (ZnO) belonging to ZnO and ethylene glycol (ZnO) also belongs to ZnO defect.

Photodegradation processes of all four samples are calculated under regulated visible light irradiation through oxidation of nitenpyram (NPT), Tetracycline (TC) and sulfamethoxazole (SMZ). Having heterojunctions makes the photodegradation process greatly improved. NPT, TC and SMZ photodegradation proficiency in 0.4 g C_3_N_4_/ZnO is 94.2%, while photocatalytic productivities for pure 0.1 g, 0.2 g and 0.3 g C_3_N_4_/ZnO are 50.1%, 56.1%, and 81.8%, respectively (Figure 7 and Figure 8). Photodegradations of NPT, TC and SMZ from Figure 6 and Figure 7 have been spotted with the institution of the photocatalyst, in the presence of visible light irradiation in Ultraviolent medium. Nevertheless, the C_3_N_4_/ZnO composites cannot categorically reduce NPT, TC and SMZ within 25 min without light irradiation. As can be detected, the solution concentration has been gently decreased in the position of maximum absorption with irradiation time, without any movements. Thus, the removals of PPCPs are deduced to follow the aromatic ring-opening pathways (Huang et al., 2016). This is in line with a report conducted by Jia and his colleagues [27]. These findings suggested that combining g-C_3_N_4_ and ZnO would improve electron and hole separation, resulting in one of the best photocatalytic efficiency candidates [28,29].

Moreover, NPT, TC and SMZ photodegradation proficiency in defect ZnO is 91.2%, while photocatalytic productivities for pure ZnO is 56.1% respectively (Figure 7 and Figure 8).

**Figure 9:**
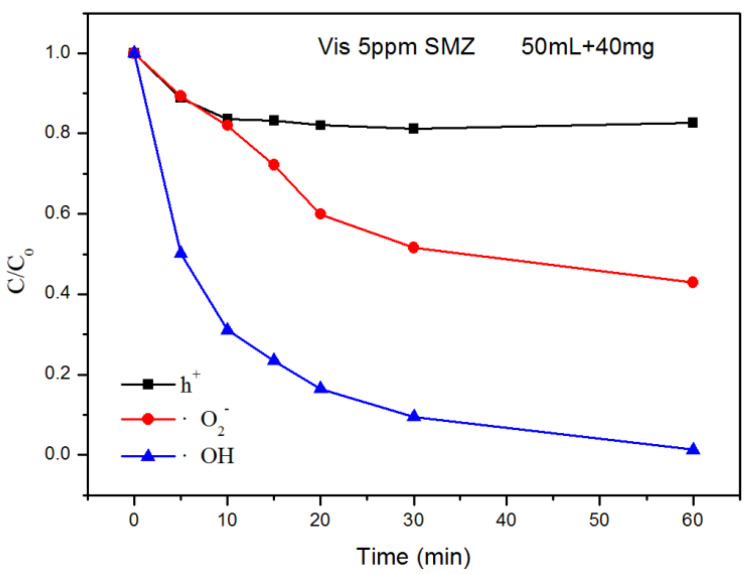
Trapping experiments of by Core shell @C_3_N_4_/ZnO catalyst with/without scavengers in the degradation of SMZ.

It is asserted that all ∙OH, ∙O^2−^ and h^+^ species may be operational in the process of photocatalytic degradation (Khan et al., 2013; Dudarev et al., 1998). The free radical trapping experiment was conducted during SMZ degradation to report the principal active substances using composite Core shell@C_3_N_4_/ZnO as the photocatalyst. During photocatalytic degradation of the SMZ, 1.0 mM of TBA (∙OH quench), EDTA (h^+^ quench) and BZQ (∙O^2−^ quench) were added as active substances capturing agents to the reaction system (Ong et al. 2016; Brindha et al., 2017; Nosaka et al. 2017; Huang et al., 2016), as shown in Figure 9. The presentation of EDTA has a negligible impact on the photocatalytic activity of Core shell @C3N4/ZnO, while the degradation of SMZ after application of TBA and BZQ has been significantly reduced. The efficiency of photocatalytic degradation has been reduced from 100% to 50%, and 14% respectively, specifying that ∙OH is the most important active species in the photocatalytic process.

## 4. Possible Mechanism

**Figure 10:**
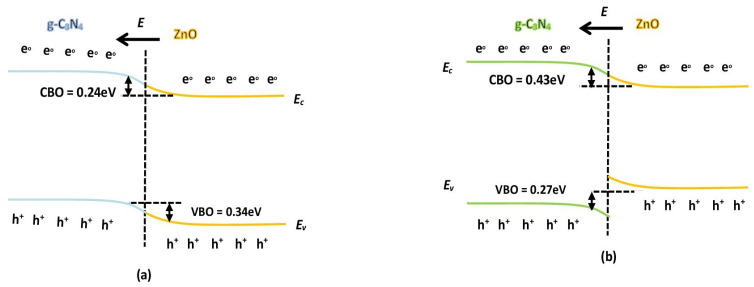
Schematic diagram revealing the photocatalytic mechanism of g-C_3_N_4_/ZnO catalyst: (**a**) conduction band of 0.24 ev and (**b**) conduction band of 0.43 eV.

A possible g-C_3_N_4_/ZnO photocatalytic mechanism is shown in Figure 10a,b, based on the above experimental findings. Fabricated excited catalysts for the visible light or sunlight—the g-C_3_N_4_/ZnO—create photo-induced electrons and holes. In the subsequent core shell device, g-C_3_N_4_ acts as a sensitizer between band edge locations, while ZnO acts as a substratum. The surface of g-C_3_N_4_ can communicate with the photo-induced electrons and react with O_2_ to form the superoxide radicals. The g-C_3_N_4_ ECB is less than the ZnO ECB; thus, ZnO can act as a sink for the produced electrons. Therefore, during photocatalytic removal of TC or NTP or SMZ, we thought that the electrons provided in the g-C_3_N_4_ CB could transfer to the ZnO CB to form a core shell photocatalyst. Holes can react openly to TC or NTP or SMZ or transfer to ZnO and contribute to the degradation of TC or NTP or SMZ. Superoxide radicals form increased load carrier separation by the consumption of electrons. g-C_3_N_4_/ZnO reduces the function of surface work with load storage capacity and serves as an active location on the semiconductor surface, greatly accelerating the transport and separation of the photo-generated load. The g-C_3_N_4_-doped ZnO sample also shows outstanding pollutant degradation.

In addition, the CB position of ZnO is less than O_2_/percent O_2_^−^’s redox potential, meaning that O_2_ could be captivated and reduced to an O_2_^−^ percentage. The remaining holes in the g-C_3_N_4_ VB are also capable of directly oxidizing TC or NTP or SMZ to the affected degradation products.

## 5. Conclusions

g-C_3_N_4_-doped ZnO composites were successfully synthesized. The light absorption shifts to a lower wavelength with the increase in the g-C_3_N_4_-doped ZnO content; the band gap energy and valence band edge potential increases the separation of the load carrier by making more electrons available for oxcarbazepine photodegradation. The photocatalyst, g-C_3_N_4_-doped ZnO, has been proven to be one of the biggest favorable candidates for the cunning and assembly of innovative photocatalysts. This study presents a better understanding of the hydrothermal synthesis techniques of g-C_3_N_4_-doped ZnO photocatalysts for better visible light responsive activity.
